# Connexin43- and Pannexin-Based Channels in Neuroinflammation and Cerebral Neuropathies

**DOI:** 10.3389/fnmol.2017.00320

**Published:** 2017-10-10

**Authors:** Denis Sarrouilhe, Catherine Dejean, Marc Mesnil

**Affiliations:** ^1^Laboratoire de Physiologie Humaine, Faculté de Médecine et Pharmacie, Université de Poitiers, Poitiers, France; ^2^Service Pharmacie, Pavillon Janet, Centre Hospitalier Henri Laborit, Poitiers, France; ^3^STIM Laboratory, ERL 7368-CNRS, Université de Poitiers, Pôle Biologie Santé, Poitiers, France

**Keywords:** connexin, pannexin, hemichannels, inflammation, neuropathies, comorbidity, gap junctions, central nervous system

## Abstract

Connexins (Cx) are largely represented in the central nervous system (CNS) with 11 Cx isoforms forming intercellular channels. Moreover, in the CNS, Cx43 can form hemichannels (HCs) at non-junctional membrane as does the related channel-forming Pannexin1 (Panx1) and Panx2. Opening of Panx1 channels and Cx43 HCs appears to be involved in inflammation and has been documented in various CNS pathologies. Over recent years, evidence has accumulated supporting a link between inflammation and cerebral neuropathies (migraine, Alzheimer’s disease (AD), Parkinson’s disease (PD), major depressive disorder, autism spectrum disorder (ASD), epilepsy, schizophrenia, bipolar disorder). Involvement of Panx channels and Cx43 HCs has been also proposed in pathophysiology of neurological diseases and psychiatric disorders. Other studies showed that following inflammatory injury of the CNS, Panx1 activators are released and prolonged opening of Panx1 channels triggers neuronal death. In neuropsychiatric diseases, comorbidities are frequently present and can aggravate the symptoms and make therapeutic management more complex. The high comorbidity between some neuropathies can be partially understood by the fact that these diseases share a common etiology involving inflammatory pathways and Panx1 channels or Cx43 HCs. Thus, anti-inflammatory therapy opens perspectives of targets for new treatments and could have real potential in controlling a cerebral neuropathy and some of its comorbidities. The purpose of this mini review is to provide information of our knowledge on the link between Cx43- and Panx-based channels, inflammation and cerebral neuropathies.

## Introduction

The innate immune system mediates inflammation for a physiological response to insult, infection, or biological stress. In most central nervous system (CNS) diseases, a common underlying factor seems to be the triggering of the inflammatory cascade with release of inflammatory cytokines (Vezzani et al., 2016). In brain, microglial cells predominantly confer innate immunity acting as resident macrophages of the CNS and represent the first line of defense against injury. However, excessive activation of microglia causes over-production of inflammatory cytokines directly affecting the CNS. Emerging evidence suggests that neurons, astrocytes and mastocytes also play important roles in neuroinflammation. Together with their strategic localization, their capacity to modulate microglial activation, their migration and activation at sites of injury demonstrate that mastocytes can initiate and/or modulate the neuroinflammatory process. Thus, understanding and control of interactions between the immune and the nervous systems might be a key for preventing most CNS diseases.

In neuropsychiatric diseases, comorbidities are frequently present and can aggravate the symptoms and make therapeutic management more complex. In migrepsy, a migraine syndrome with aura, an epileptic seizure follows migraine attack in a way suggesting that one would follow the other. A complex of comorbidities including migraine, major depression disorder (MDD) and suicide is also observable (Nye and Thadani, 2015). Depressive episodes are the most common comorbidity in epilepsy, affecting between 11% and 62% of epileptic patients (Błaszczyk and Czuczwar, 2016). A meta-analysis confirmed that psychiatric comorbidities are more common in patients with treatment-resistant epilepsy (Scott et al., 2017). In patients with epilepsy, the proportion of psychotic disorders is higher than in the non-epileptic population, but the increased risk of schizophrenia varies according to the study (Bakken et al., 2014). Interestingly, a link between epilepsy, autism spectrum disorder (ASD), depression and the brain inflammatory pathways was revealed (Mazarati et al., 2017). In the early stages of Alzheimer’s disease (AD), patients may have generalized convulsive seizures but also partial epilepsies located in the frontal or temporal lobe (Cretin et al., 2017). In addition, psychiatric comorbidities such as depression, schizophrenia and bipolar disorders may be severe, prodromal and predispose to the development of AD (Garcez et al., 2015).

A common part between all these brain pathologies/comorbidities could be inflammation in which gap junction proteins seem to be involved. Such an involvement is not surprising when considering that gap junctions and their structural proteins, the connexins (Cx), are very present in the CNS in which cells have to be efficiently connected for treating incoming information from the body and its environment and controlling consequently adapted physiological responses. And indeed, several CNS cell functions (electrical synapses in neurons, ionic and neurotransmitter buffering by astrocytes, propagation of astrocytic Ca^2+^ waves, myelin stabilization in oligodendrocytes, etc.) appeared to be supported by gap-junctional intercellular communication (GJIC; Giaume and Venance, 1998; Deans et al., 2001; Eugenin et al., 2012; Georgiou et al., 2017). Such crucial roles may explain the presence of 11 out 21 Cx isoforms in human CNS that are differently dispatched between astrocytes, oligodendrocytes, microglia and neurons (Giaume and Liu, 2012). However, more recently, it appeared that Cxs mediate communication between intracellular and extracellular compartments by forming non-juxtaposed hemichannels (HCs). In brain, this activity is mostly observed for Cx43 under various stimuli that permit the release of transmitters like glutamate and ATP (Giaume et al., 2013). Such activity is shared by pannexins (Panx) which are distant homologs of Cxs unable to form gap junctions but transmembrane channels. Interestingly, the different forms of communication permitted by Cxs and Panxs are involved in CNS inflammation with various effects that depend on the communication type. For instance, extracellular factors such as pro-inflammatory cytokines (IL-6, IL-1β, TNF-α) which are liberated by microglia in case of inflammation inhibit Cx43-mediated GJIC in astrocytes whereas their HCs are activated (Retamal et al., 2007). Cx43 HCs are indeed open during inflammation, contributing to the activation of the inflammasome pathway and its spread to neighboring cells (Kim et al., 2016). Similar action has been observed for Pannexin1 (Panx1), which activates inflammasome in astrocytes and is involved in ischemic injury (Bennett et al., 2012; Makarenkova and Shestopalov, 2014). All these observations elicit Cx43 and Panx1 as therapeutical targets whose inhibition could decrease inflammation in CNS. On this aspect, recent data identifying inhibitors of Cx43 HCs (tonabersat) or Panx1 channels (probenecid) to prevent inflammasome activation and damage in the CNS are encouraging (Jian et al., 2016; Kim et al., 2017).

The purpose of this mini review is to provide information on the link between Cx43- and Panx-based channels, inflammation and cerebral neuropathies.

## Migraine with Aura

Neuroimaging and experimental studies suggest that cortical spreading depression (CSD), a slow wave of neuronal and glial depolarization, triggers migraine aura, activates the trigeminovascular system and is possibly responsible for migraine headache (Charles and Baca, 2013; Sarrouilhe et al., 2014). Recently, Karatas et al. (2013) elucidated the cellular and molecular mechanisms linking CSD induction and activation of the trigeminovascular system in mice, involving a parenchymal inflammatory process. Upon CSD induction, neuronal Panx1 channels transiently open with subsequent activation of a multiprotein complex (inflammasome) that mediates the innate inflammatory response. The initiation of the inflammatory response involves the proteolytic activation of caspase-1 and a release of high-mobility group box 1 (HMGB1) and interleukin-1β (IL-1β; Silverman et al., 2009; Karatas et al., 2013). Subsequently, in astrocytes forming the *glia limitans*, nuclear factor KappaB (NF-κB) is activated and translocated to the nucleus and nitric oxide synthase (iNOS) and cyclooxygenase-2 (COX2) are induced. Then, cytokines, prostanoids and NO, released in the subarachnoid space, promote an activation of the perivascular nociceptive trigeminal ending in *pia mater* that may induce headache. Pharmacological inhibition of neuronal Panx1 channels by carbenoxolone abolishes the inflammatory signaling cascade, perivascular nociceptive trigeminal activation and reduces CSD-induced mast cell degranulation. Even if these results suggest that neuronal Panx1 channels are a link between stressed neurons and subsequent inflammatory pathways, further investigations taking recourse to Panx1 knock-out mice are necessary to validate these findings (Karatas et al., 2013).

## Alzheimer’s Disease

Several cell types would play a role in neuroinflammation observed in AD. Among them, microglial cells are known to contribute to the chronic inflammation state observed in AD. Increased levels of inflammatory mediators detected in brains from AD patients contribute to disease progression and severity (Heneka et al., 2015). *In vitro* treatment with active fragment of the amyloid beta peptide induces microglial Cx43 HC and Panx1 channel opening. Activated microglia releases pro-inflammatory cytokines that contribute to the amyloid peptide-induced Cx43 HCs and Panx1 channels opening in astrocytes (Orellana et al., 2011a). In reactive astrocytes, Cx43 is the main HC contributor whereas Panx1 channels are restricted to astrocyte subpopulation contacting amyloid plaques (Yi et al., 2016). Both microglia and astrocytes could release gliotransmitters (ATP/glutamate) through HCs, resulting in neuronal Cx36 HCs and Panx1 channels opening that triggers neuronal death (Orellana et al., 2011b; Koulakoff et al., 2012). Mastocyte secretory granules contain pro-inflammatory mediators released in the extracellular milieu via a Ca^2+^ influx upon activation. When the progression of AD is studied in a murine model of AD (APPswe/PS1dE9 mice), the number of mastocytes in cortical and hippocampal areas early increases. Moreover, patients with AD present mastocytes near their amyloid plaques. Acute treatment with amyloid beta peptide induces rapid degranulation of cultured mastocytes via a Panx1 channel-dependent Ca^2+^ influx and this response is prevented by Panx1 blockers. In brain mastocytes, acute treatment with amyloid beta peptide also induces activity of Panx1 channels and Cx43 HCs, an effect that is associated with enhanced histamine release. In the presence of amyloid plaques, brain mastocytes of APPswe/PS1dE9 mice show high Panx1 channel and Cx43 HC activity (Harcha et al., 2015). Thus, channel response of mastocytes to amyloid peptide treatment seems to occur earlier than microglia, astrocyte and neuron (Orellana et al., 2011b; Harcha et al., 2015). Therefore, mastocytes might be brain cells that play a critical role in the onset and progression of AD by early sensing amyloid peptide, releasing pro-inflammatory molecules and recruiting other cells to the neuroinflammatory response.

## Parkinson’s Disease (PD)

Chronic neuroinflammation is a characteristic of PD. Glial cell activation and increased pro-inflammatory molecules are observed in brains of PD patients and animal models. The chronic release of pro-inflammatory cytokines exacerbates the motor symptoms of PD resulting from dopaminergic neuron degeneration in the *substancia nigra pars compacta* (Wang et al., 2015). Cx43 upregulation has been identified in the striatum of rodent models of PD and in cultured astrocytes stimulated with rotenone (Xie et al., 2015). Moreover, gastrodin, a constituent of a Chinese herbal medicine, ameliorates PD by downregulating astrocytic Cx43 (Wang et al., 2013). The neuronal build-up of protein aggregates containing alpha-synuclein (ASN) and their release to the extracellular space are considered to be responsible for the propagation of neurodegeneration in the brain of advanced PD patients (Wang et al., 2013). A recent study showed that extracellular ASN neurotoxicity is mediated by the P2X7 receptor signaling complex. Treatment of neuroblastoma cells and rat synaptoneurosomes with exogenous ASN activated P2X7 receptors leading to Panx 1 recruitment responsible for ATP release that could lead to neurotoxicity (Wilkaniec et al., 2017).

## Major Depressive Disorder

Whereas many studies suggested astrocytic gap junction dysfunction to be part of MDD etiologies (Sarrouilhe and Dejean, 2015), the respective roles of GJIC and Cx43 HCs are not elucidated (Quesseveur et al., 2015; Jeanson et al., 2016). In a functional study made in cortical and striatal mouse astrocytes, tested antidepressants were shown to exert different effects on Cx43 GJIC and HC activities (Jeanson et al., 2016). Indeed, even if they exhibit opposed effects on GJIC within a same therapeutic class, all tested drugs inhibit Cx43 HCs (Jeanson et al., 2016). Experimental and clinical data point to a role for inflammation in the development of MDD (Capuron and Miller, 2011). In MDD patients, expression of pro-inflammatory cytokines such as TNF-α and IL1-β are increased and correlate with the MDD severity. Under neuroinflammation conditions, microglia is primary stimulated and releases these cytokines that open Cx43 HCs in astrocytes while no change is observed in GJIC (Abudara et al., 2015). Moreover, several antidepressants are known to inhibit the production of these cytokines. Recently, a study made on patients with mastocytosis, a rare accumulation and activation of mast cells in various tissues, demonstrates their possible involvement in inflammation-induced depression, confirming their implication in inflammatory diseases (Georgin-Lavialle et al., 2016). On the other hand, MDD and AD, frequently co-occur and it was suggested that depression increases the risk of subsequent AD. Globally, although the data is scattered, as in the case of AD, increased mastocytes HC activity might be an early player in the inflammation pathways linked to MDD. It is clear that further research is necessary to decipher the cascade of events taking place between mastocytes activation, astrocytic gap junctions and Cx43 HC involvement, and neuronal dysfunction.

## Autism Spectrum Disorder

ASD is a complex group of disorders associated with aberrant chemical synaptic transmission and plasticity (Zoghbi and Bear, 2012). Young patients with ASD have a surplus of chemical synapses due to a slowdown process involving microglia that early eliminates about half of cortical synapses (Tang et al., 2014). Few data are available about the involvement of Cx43 in ASD. Studies using conditional Cx43 knockout mice show that Cx43 is important for neurodevelopment (Wiencken-Barger et al., 2007). In postmortem brain tissues of ASD patients, Cx43 expression is increased in superior frontal cortex, a region which dysfunction may be responsible for cognition deficit observed in this neuropathy (Fatemi et al., 2008). Moreover, growing evidence indicates that the two forms of synapses interact during brain development and could contribute, together, to ASD (Miller et al., 2015; Pereda, 2015). Several studies have shown that neuroinflammation plays an important role in ASD and that mastocytes are overactivated. In ASD, pre-natal or early post-natal inflammatory and infectious processes correlate to neurodevelopmental dysfunction (Wang et al., 2014). Perinatal mastocytes activation by various triggers (infectious, stress-related, environmental, allergic) with a subsequent release of pro-inflammatory and neurotoxic molecules can contribute to brain inflammation in ASD pathogenesis through a cascade of events involving glial cells (microglia, astrocytes, oligodendrocytes), neurons, Cx43 HCs and Panx channels (Orellana et al., 2011b; Aguirre et al., 2013). It was also proposed that loss of Cx43 in the enteric glial cells contribute to brain inflammation in ASD by inducing disturbances in the gut-brain axis (Grubišc and Parpura, 2015).

## Epilepsy

Experimental approaches demonstrated a prominent role of glial cells, activated during infectious and non-infectious causes of inflammation, in the mechanisms of seizure precipitation and recurrence. Both causes of inflammation share common pathways with activation of microglia and astrocytes releasing pro-inflammatory mediators that perturb glioneuronal communication and have proictogenic properties (Vezzani et al., 2016). Although GJs, Cx and Panx are related to the pathophysiology of epilepsy, their precise involvement is not elucidated. Besides interneuronal GJIC, inter-glial GJIC appears important for seizure generation. However, there is evidence from most animal models and patients studies that Cx43 expression increases in glia but not in neurons, opening the question on the roles of glia in seizure generation. A systematic analysis of the literature reveals significant changes in expression of the astrocytic Cx43 and Panx1 in an *in vitro* mouse seizure model and an increase of Panx1-2 expression in animal and human epileptic tissues (Mylvaganam et al., 2014). Using various approaches and Panx1-deleted mice, Panx1 channels (from glia and/or neurons) were proposed to contribute to *status epilepticus in vivo*. The data of this study are consistent with the following model: (1) the intense neuronal activity elevates extracellular K^+^; (2) Panx1 channels are activated; (3) ATP is released; and (4) P2X receptors are activated leading to neuronal hyperactivity and this positive feedback mechanism amplifies seizures (Santiago et al., 2011). Antiepileptic drugs tested in an astroglia/microglia co-culture model of inflammation did not alter Cx43 expression (Dambach et al., 2014). In contrast, tonabersat, a compound active against neuronal hyperexcitability and neurogenic inflammation that was proposed in the treatment for epilepsy and as a prophylactic treatment for migraine with aura, prevents inflammatory damage in the CNS by blocking Cx43 HCs (Kim et al., 2017).

## Other Pathologies Potentially Mediated by Cx43 and Panx-Based Channels Activity

A recent analysis of all available data on neuroinflammation in postmortem brains of schizophrenia patients revealed variable results in astrocytic and microglial markers, glial cell density and pro-inflammatory cytokine concentration (Trépanier et al., 2016). Moreover, prenatal exposure to inflammatory conditions (i.e., LPS-exposed dams) revealed that the release of pro-inflammatory cytokines (IL-1β/TNF-α) and ATP through the activation of astrocytic Cx43 HCs and Panx1 channels results in an astrocyte-neuron crosstalk with paracrine activation of neuronal P2X7 receptors, Panx1 channels and a subsequent increased neuronal death. All these data suggest that prenatal infections could contribute to the development of neuropsychological disorders in children including schizophrenia (Avendaño et al., 2015). However, a recent work does not support a major contribution of Panx1-3 to disease risk of schizophrenia (Gawlik et al., 2016).

In the case of postmortem bipolar disorder patients, a post-mortem study revealed in their frontal cortex an increase of excitotoxicity and neuroinflammatory markers. This upregulation might explain the neurodegenerative component of bipolar disorder, with cell death, brain atrophy and cognitive decline. Markers of astrocyte and microglial activation are also upregulated in frontal cortex from bipolar disorder patients (Rao et al., 2010). Interestingly, in light of the involvement of mastocytes in some other psychiatric neuropathies, bipolar disorder is a psychiatric symptom observed in mast cell activation syndrome (Afrin, 2014). A dysregulation of Cx expression in the astrocytic syncytium can cause an imbalance in glutamatergic tripartite synapses and was proposed to be responsible for the pathophysiology of bipolar disorder (Mitterauer, 2011).

## Conclusion

Researching links between inflammation, neurological diseases and psychiatric disorders with Cx and Panx channels is at a very nascent stage but has the potential to improve our understanding of these diseases to establish effective therapeutics. For some diseases like migraine with aura or AD, a signaling cascade is proposed while for others the data are too sparse. Common inflammatory pathways can explain the high comorbidity between some neuropathies. Thus, anti-inflammatory therapy could have real potential in controlling a cerebral neuropathy and some of its comorbidities (Mazarati et al., 2017). In this line, tonabersat, a compound active against neurogenic inflammation proposed in the treatment for epilepsy and as a prophylactic treatment for migraine with aura, two frequent comorbidities, prevents inflammatory damage in the CNS by blocking Cx43 HCs (Kim et al., 2017). Cx43 HCs can open in response to injury or inflammatory factors and are thus implicated in brain neuropathies, especially through the inflammasome pathway. Modulating the Cx43 HCs opening can prevent tissue damage arising from excessive and uncontrolled inflammation (Kim et al., 2016).

Panx1 channels drive inflammation by the regulation of inflammasome, the release of pro-inflammatory cytokines and the activation and migration of leukocytes (Crespo Yanguas et al., 2017). They also facilitate neuronal cell death that potentially implicates them in neurodegenerative disorders (Shestopalov and Slepak, 2014). Blockers of Panx1 channels are not highly selective as they also block GJIC, and many deleterious side effects limit their pharmacological potential.

The development of new therapeutic tools to inhibit selectively Panx1 channels and Cx43 HCs in cell subpopulations of the CNS will undoubtedly make them a promising target for anti-inflammatory therapy that could have real potential in prevention or delay of neurological diseases, psychiatric disorders and some of their comorbidities. However, such a strategy should not only focused on Cx43 but also to other highly expressed Cxs in the CNS like Cx36 which appears to be involved in several neuronal injuries (autism, ischemia, retina, etc.; Welsh et al., 2005; Bargiotas et al., 2012; Ivanova et al., 2016). Another main goal for the future is to define the respective role of Cx HCs and Panx1 channels in neuroinflammation and cerebral neuropathies. Among the few studies in this area, it was shown in reactive astrocytes of a mouse model of AD that their respective contribution seems to depend on the local environment context (Yi et al., 2016).

## Author Contributions

DS wrote and edited the manuscript, conceived and designed the major ideas developed in the manuscript. MM and CD have made substantial, direct and intellectual contribution to the work. All authors read and approved the final manuscript.

## Conflict of Interest Statement

The authors declare that the work was conducted in the absence of any commercial or financial relationships that could be construed as a potential conflict of interest.
